# Case report: a rare case of diffusely metastatic *BRAF* V600E-mutated colorectal cancer with concomitant infiltration of the skin and parotid gland

**DOI:** 10.3389/fonc.2025.1512000

**Published:** 2025-01-28

**Authors:** Aristeidis E. Boukouris, Ioannis Kokkinakis, Elias Drakos, Maria Sfakianaki, Maria Tzardi, Dimitrios Mavroudis, John Souglakos

**Affiliations:** ^1^ Department of Medical Oncology, University General Hospital of Heraklion, Heraklion, Greece; ^2^ Department of Pathology, University General Hospital of Heraklion, Medical School, University of Crete, Heraklion, Greece; ^3^ Laboratory of Translational Oncology, Medical School, University of Crete, Heraklion, Greece

**Keywords:** case report, colorectal cancer, BRAF V600E mutation, skin disease, parotid gland infiltration, fulminant metastatic behavior

## Abstract

Metastastic disease affects up to 50% of colorectal cancer (CRC) patients and is associated with particularly poor outcomes in the presence of the *BRAF* V600E mutation. Herein, we report a patient with initial diagnosis of stage IIIc CRC, who presented during follow-up (adjuvant phase) with dysphagia, left-sided lagophthalmos and multiple skin nodules. The ensuing work-up revealed disseminated metastatic disease from the primary CRC, which was *BRAF* V600E-mutated (retrospective tissue analysis), affecting, besides the lungs, multiple uncommon sites, such as the skin and parotid gland. The patient’s rapid disease progression did not allow for any therapeutic interventions. This is only the second report of concomitant metastatic infiltration of the skin and parotid gland by CRC, and the first with a documented molecular background of *BRAF* V600E mutation. *BRAF* V600E-mutated CRC can follow an aggressive and often unpredictable clinical course in the metastatic setting that physicians should be aware of, and the molecular profile of the tumor at diagnosis could be useful for comprehensive and timely management.

## 1 Introduction

Colorectal cancer is the 4^th^ most common cancer worldwide and represents the 2^nd^ leading cause of cancer-related mortality (1). As many as 50% of CRC patients eventually go on to develop metastatic disease (*de novo* or secondary), predominantly affecting the liver, lung and peritoneum (in order of frequency). The *BRAF* V600E mutation (representing 95% of all CRC-related *BRAF* mutations) is only encountered in about 8% of metastatic CRC (mCRC) patients (2, 3), however it is associated with poor prognosis and a highly aggressive behavior, including resistance to currently available therapies and predilection for metastasis to distant lymph nodes and the peritoneum (4, 5). In this report, we describe a rare case of *BRAF* V600E-mutated CRC with secondary metastatic disease concomitantly affecting the skin and parotid gland.

## 2 Case presentation

**Figure 1:**
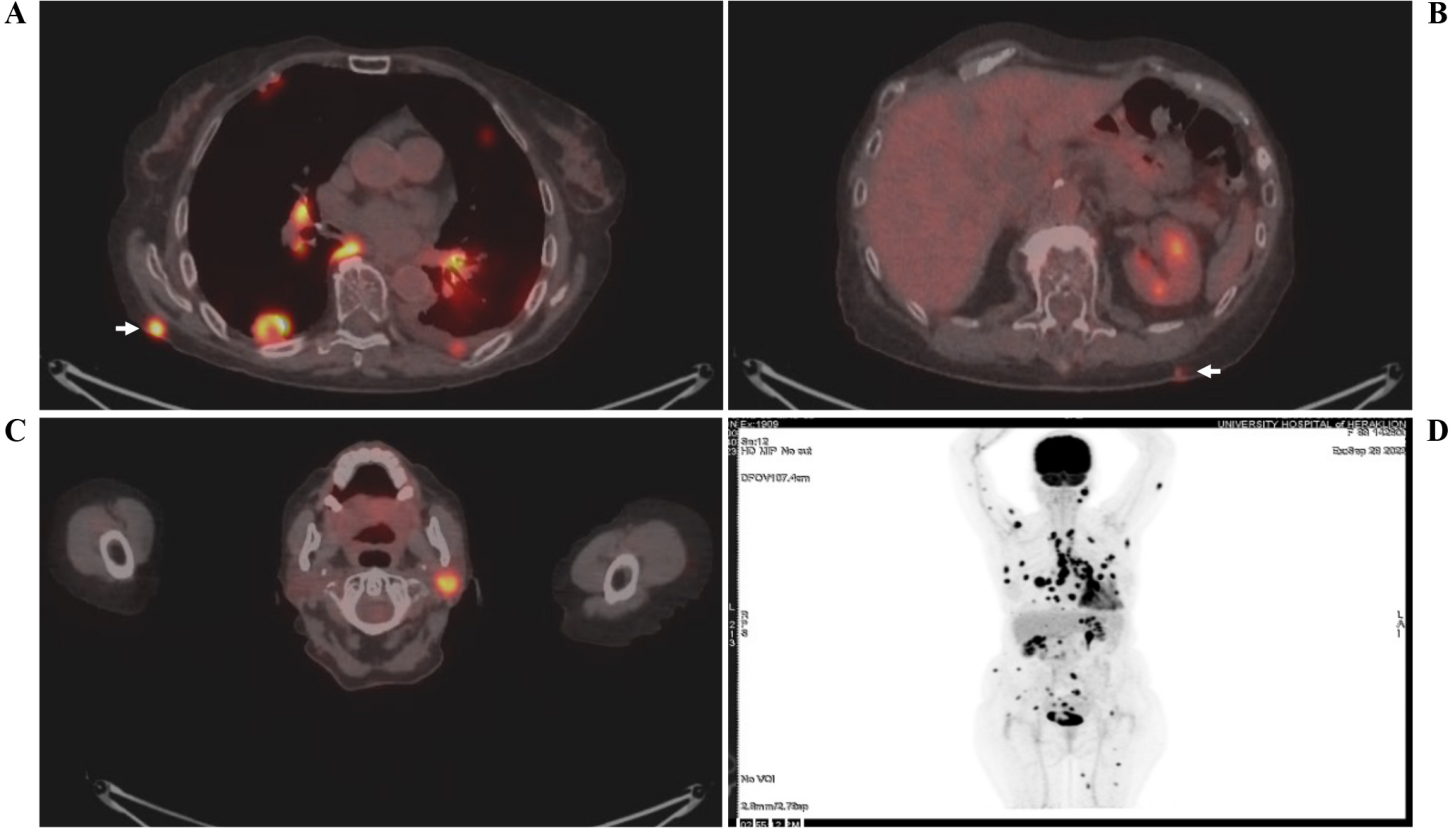
^18^F-FDG-PET/CT scan showing hypermetabolic uptake in the **(A, B)** skin (white arrows) and **(C)** left parotid gland. A whole-body scan **(D)** is also shown. The patient’s name and date of birth have been omitted for confidentiality reasons.

**Figure 2:**
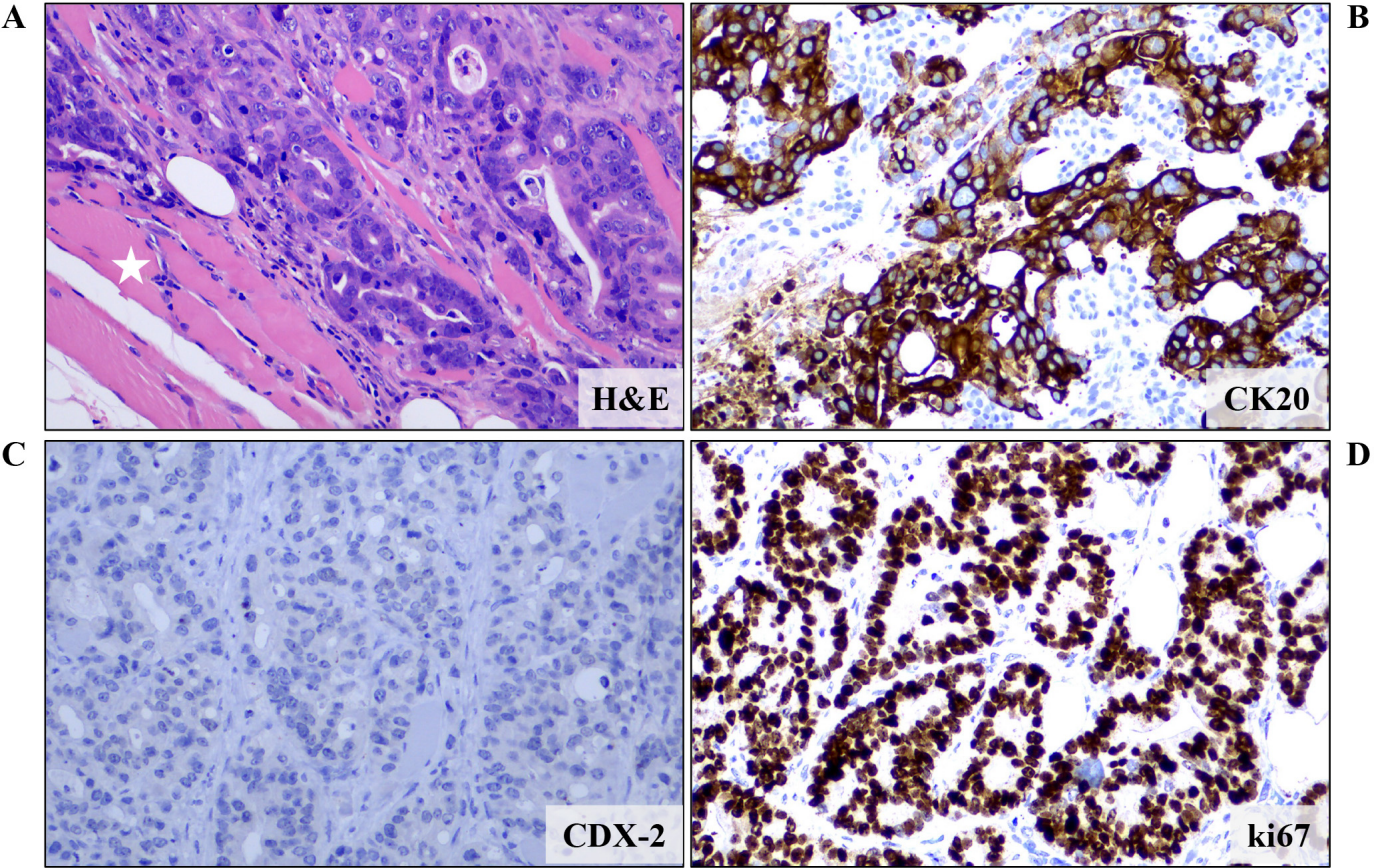
Microscopic examination of a biopsy from a skin lesion revealed infiltration of fat and striated muscle tissue (white asterisk) from an enteric type adenocarcinoma (tubular and cribriform pattern) **(A)** (H&E staining). Immunohistochemical analysis showed that the neoplastic cells expressed **(B)** CK20 but not **(C)** CDX-2, CK7 and TTF-1 (data not shown for CK7 and TTF-1). These features, combined with the patient’s history, were more compatible with a metastatic colorectal carcinoma. **(D)** Proliferation rate, as expressed by ki67 positivity, was almost 100%. Images are shown at 200x magnification. DAB as chromogen and hematoxylin as counterstain. CK, Cytokeratin; H&E, Hematoxylin and Eosin.

An 83-year-old female patient (birthplace and residence: Crete, Greece) and former smoker (50 pack-years) with a past medical history of arterial hypertension and a family history of a first-degree relative with CRC at an advanced age, was recently diagnosed with stage IIIc (pT3N1b) caecal adenocarcinoma. Following right colectomy, the patient was placed on adjuvant therapy based on the Roswell Park regimen. After uneventful completion of the first cycle, the second cycle was interrupted midway due to GI disturbances and the patient feeling unwell. She presented to the outpatient clinic two months later due to intractable fatigue, along with dysphagia and left-sided lagophthalmos of recent onset. Clinical examination confirmed the dysphagia and revealed left-sided Bell’s palsy. Intriguingly, multiple firm nodules were noted on the trunk and extremities, along with a palpable firm nodule at the anatomic position of the left parotid gland. Routine complete blood count and basic metabolic panel did not reveal any abnormalities. However, the tumor marker CEA was significantly increased since it was last measured (8,1 ng/ml *vs.* < 1,73 ng/ml), raising suspicion of disease progression. Further investigation with ^18^F-FDG-PET/CT scan revealed hypermetabolic foci in the skin and left parotid gland (potentially accounting for the observed Bell’s palsy) (**Figure 1**), as well as multiple other sites (lung, bones, muscles, peritoneum and multiple lymph nodes (cervical, mediastinal, portal)). Molecular analysis of the archival primary tumor tissue revealed the presence of the *BRAF* V600E mutation. Biopsies of the skin lesions revealed infiltration by a low-grade enteric type adenocarcinoma, consistent with the morphologic characteristics of the primary tumor (**Figure 2A-C**). The metastatic origin of the lesions was further confirmed by detection of the *BRAF* V600E mutation via genetic testing. Notably, metastatic cancer cells exhibited an extremely high proliferation rate (ki67 positivity: 95%) (**Figure 2D**).

A few days after admission, the patient developed acute, severe respiratory insufficiency requiring high oxygen supply (Venturi mask). Besides signs of aspiration pneumonia, repeat chest X-ray revealed a diffuse opacification of the left hemithorax, consistent with a new, massive left-sided pleural effusion. Cytologic analysis of the fluid was positive for the presence of malignant epithelial cells. Despite fluid drainage and other supportive measures, the patient rapidly deteriorated, eventually succumbing to her disease.

**Figure 3:**
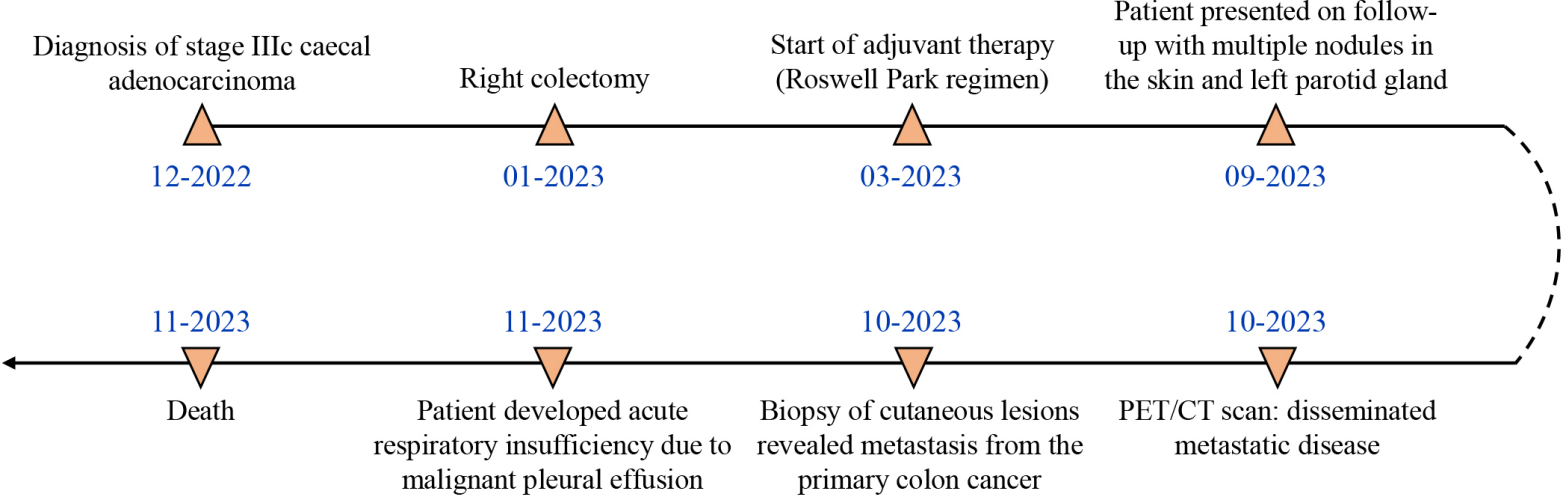
Timeline of events and case progression.

The timeline of events and case progression with relevant data are presented in **Figure 3**.

## 3 Discussion

We present a patient with recently diagnosed stage IIIc CRC who relapsed with fulminant metastatic disease affecting many organs, among others the skin and parotid gland. Molecular analysis of the archival tumor tissue revealed the presence of the *BRAF* V600E mutation. No *HER2* amplification and *KRAS* (exon 2, 3 and 4) mutations were detected. Testing of the MSI status, using PCR-based fragment analysis, was negative for microsatellite instability (20-30% of *BRAF* V600E-mutated CRCs are dMMR (6)). Presence of the *BRAF* V600E mutation represented the dominant oncogenic driver, accounting for the aggressive behavior of the disease. Consistent with the very high proliferation rate of the metastatic cancer cells and the multi-organ involvement, our patient’s condition and performance status rapidly deteriorated, precluding any therapeutic manipulations.

Our group has previously described the clinical aspects of the *BRAF* V600E mutation in CRC, which has been correlated with rapidly progressive multimetastatic disease, poor performance status, advanced age, peritoneal disease and low probability of secondary metastasectomy (7). Furthermore, a later Danish population-based study in 448 CRC patients (of which 30 carried the *BRAF* V600E mutation), associated the presence of the *BRAF* V600E mutation with increased risk of skin metastases (8). Our case confirms these observations and is the first to describe concomitant malignant infiltration of both the skin and parotid gland from CRC with documented *BRAF* V600E mutation. The rarity of similar cases, together with the lack of complete molecular characterization of the (primary or metastatic) tumor specimens (9) preclude potential identification of more specific clinical features of patients with *BRAF* V600E-mutated CRC who eventually develop metastatic infiltration of the skin and parotid gland.

The molecular basis behind the predilection of *BRAF* V600E-mutated CRC cells for invasion of the skin (and other unusual sites, such as the parotid gland), remains elusive. Of note, enrichment of the *BRAF* V600E mutation in cutaneous metastatic disease has also been documented for other types of cancer (lung cancer, papillary thyroid carcinoma), which only rarely affect the skin (10, 11). This suggests the possibility of a common underlying mechanism in *BRAF* V600E-mutated cells, perhaps related to downstream activation of hypoxia-inducible factors (*HIFs*) (12–14), which allows survival of metastatic cells in the mildly hypoxic skin microenvironment (15).

We also propose that assessment of the *BRAF* V600E mutation should be considered at the time of diagnosis, regardless of disease stage, since it may modify the treatment intent (palliative or curative) and influence the treatment strategy. Our approach is supported by a recent Italian expert opinion statement, which supports *BRAF* mutation testing not only in the metastatic setting but also in high-risk stage III CRC patients (16). This is because knowledge of the *BRAF* status and the prognostic significance of *BRAF* mutations could inform physicians about the strong possibility of early (adjuvant) treatment failure, as previously demonstrated in several cohorts (17, 18). Moreover, in case of disease recurrence, it could facilitate rapid-decision making and initiation of the appropriate treatment modality. As a matter of fact, targeted anti-*BRAF* agents have now largely replaced chemotherapy-based regimens for the treatment of *BRAF* V600E-mutated mCRC. Very recently, encorafenib (a *BRAF* V600 inhibitor) and cetuximab (an anti-*EGFR* monoclonal antibody), in combination with mFOLFOX6, received accelerated FDA approval in the 1^st^ line treatment of *BRAF* V600E-mutated mCRC based on the phase III BREAKWATER trial. Updated results of this trial are expected within the next few weeks, and they may change the standard of care in the 1^st^ line treatment. In the 2^nd^ line, mCRC patients with the *BRAF* V600E mutation can be effectively treated with the combination of encorafenib and cetuximab (19). Real-world data have confirmed the efficacy of this regimen (objective response rate, ORR: 32%) (20). Other combination regimens, such as dabrafenib (a selective *BRAF* inhibitor) and trametinib (a selective *MEK* inhibitor) have also been formerly tested, however with poorer outcomes (ORR: 12%).

In conclusion, our rare case confirms the aggressive phenotype conferred to CRC by the *BRAF* V600E mutation, and highlights the potential of this CRC subtype for concomitant metastatic infiltration of many non-classical target sites. Oncologists handling such patients should be aware of the unpredictable and potentially fulminant metastatic behavior of *BRAF* V600E-mutated CRCs, and that testing for *BRAF* V600E at the time of diagnosis may offer the opportunity for effective treatment earlier during the course of the disease, thus optimizing patients’ management.

## Data Availability

The original contributions presented in the study are included in the article/Supplementary Material. Further inquiries can be directed to the corresponding author.

## References

[1] Siegel RL Miller KD Wagle NS Jemal A . Cancer statistics, 2023. CA Cancer J Clin. (2023) 73:17–48. doi: 10.3322/caac.21763 36633525

[2] Saridaki Z Papadatos-Pastos D Tzardi M Mavroudis D Bairaktari E Arvanity H . BRAF mutations, microsatellite instability status and cyclin D1 expression predict metastatic colorectal patients’ outcome. Br J Cancer. (2010) 102:1762–8. doi: 10.1038/sj.bjc.6605694 PMC2883698 20485284

[3] Souglakos J Philips J Wang R Marwah S Silver M Tzardi M . Prognostic and predictive value of common mutations for treatment response and survival in patients with metastatic colorectal cancer. Br J Cancer. (2009) 101:465–72. doi: 10.1038/sj.bjc.6605164 PMC2720232 19603024

[4] Tabernero J Ros J Élez E . The evolving treatment landscape in BRAF-V600E–mutated metastatic colorectal cancer. Am Soc Clin Oncol Educ Book. (2022) 42):254–63. doi: 10.1200/EDBK_349561 35503983

[5] Zhi J Jia XJ Yan J Wang HC Feng B Xing HY . BRAF(V600E) mutant colorectal cancer cells mediate local immunosuppressive microenvironment through exosomal long noncoding RNAs. World J Gastrointest Oncol. (2021) 13:2129–48. doi: 10.4251/wjgo.v13.i12.2129 PMC8713331 35070047

[6] Piercey O Tie J Hollande F Wong HL Mariadason J Desai J . BRAF(V600E)-mutant metastatic colorectal cancer: current evidence, future directions, and research priorities. Clin Colorectal Cancer. (2024) 23:215–29. doi: 10.1016/j.clcc.2024.04.004 38816264

[7] Saridaki Z Tzardi M Sfakianaki M Papadaki C Voutsina A Kalykaki A . BRAFV600E mutation analysis in patients with metastatic colorectal cancer (mCRC) in daily clinical practice: correlations with clinical characteristics, and its impact on patients’ outcome. PloS One. (2013) 8:e84604. doi: 10.1371/journal.pone.0084604 24367680 PMC3867547

[8] Christensen TD Palshof JA Larsen FO Poulsen TS Hogdall E Pfeiffer P . Associations between primary tumor RAS, BRAF and PIK3CA mutation status and metastatic site in patients with chemo-resistant metastatic colorectal cancer. Acta Oncol. (2018) 57:1057–62. doi: 10.1080/0284186X.2018.1433322 29380640

[9] Nasti G Facchini G Caraglia M Franco R La Mura A Staiano M . Concomitant occurrence of facial cutaneous and parotid gland metastases from rectal cancer after preoperative chemoradiotherapy. Onkologie. (2007) 30:324–6. doi: 10.1159/000102538 17551257

[10] Erickson LA Jin L Nakamura N Bridges AG Markovic SN Lloyd RV . Clinicopathologic features and BRAF(V600E) mutation analysis in cutaneous metastases from well-differentiated thyroid carcinomas. Cancer. (2007) 109:1965–71. doi: 10.1002/cncr.v109:10 17387744

[11] Wang X Wang H Jia B He F Yuan Y Zhang W . Cutaneous metastasis as the first presentation of non-small-cell lung cancer with a BRAF mutation: A case report. Onco Targets Ther. (2020) 13:13143–9. doi: 10.2147/OTT.S282593 PMC7767729 33380804

[12] Kikuchi H Pino MS Zeng M Shirasawa S Chung DC . Oncogenic KRAS and BRAF differentially regulate hypoxia-inducible factor-1alpha and -2alpha in colon cancer. Cancer Res. (2009) 69:8499–506. doi: 10.1158/0008-5472.CAN-09-2213 PMC2811371 19843849

[13] Kumar SM Yu H Edwards R Chen L Kazianis S Brafford P . Mutant V600E BRAF increases hypoxia inducible factor-1alpha expression in melanoma. Cancer Res. (2007) 67:3177–84. doi: 10.1158/0008-5472.CAN-06-3312 17409425

[14] Zerilli M Zito G Martorana A Pitrone M Cabibi D Cappello F . BRAF(V600E) mutation influences hypoxia-inducible factor-1alpha expression levels in papillary thyroid cancer. Mod Pathol. (2010) 23:1052–60. doi: 10.1038/modpathol.2010.86 20473281

[15] Swartz HM Flood AB Schaner PE Halpern H Williams BB Pogue BW . How best to interpret measures of levels of oxygen in tissues to make them effective clinical tools for care of patients with cancer and other oxygen-dependent pathologies. Physiol Rep. (2020) 8:e14541. doi: 10.14814/phy2.14541 32786045 PMC7422807

[16] Malapelle U Angerilli V Intini R Bergamo F Cremolini C Grillo F . Detecting BRAF mutations in colorectal cancer in clinical practice: An Italian experts’ position paper. Crit Rev Oncol Hematol. (2024) 206:104574. doi: 10.1016/j.critrevonc.2024.104574 39581242

[17] Rasola C Laurent-Puig P Andre T Falcoz A Lepage C Aparicio T . Time to recurrence and its relation to survival after recurrence in patients resected for stage III colon cancer. Eur J Cancer. (2023) 194:113321. doi: 10.1016/j.ejca.2023.113321 37797388

[18] Zhu L Dong C Cao Y Fang X Zhong C Li D . Prognostic role of BRAF mutation in stage II/III colorectal cancer receiving curative resection and adjuvant chemotherapy: A meta-analysis based on randomized clinical trials. PloS One. (2016) 11:e0154795. doi: 10.1371/journal.pone.0154795 27138801 PMC4854379

[19] Tabernero J Grothey A Van Cutsem E Yaeger R Wasan H Yoshino T . Encorafenib plus cetuximab as a new standard of care for previously treated BRAF V600E-mutant metastatic colorectal cancer: updated survival results and subgroup analyses from the BEACON study. J Clin Oncol. (2021) 39:273–84. doi: 10.1200/JCO.20.02088 PMC8078423 33503393

[20] Gallois C Bergen ES Auclin É Pernot S Higué J Trouilloud I . Efficacy and safety of the combination of encorafenib/cetuximab with or without binimetinib in patients with BRAF V600E-mutated metastatic colorectal cancer: an AGEO real-world multicenter study. ESMO Open. (2024) 9:103696. doi: 10.1016/j.esmoop.2024.103696 39255538 PMC11415680

